# Ten Years of Quality Monitoring of Abdominal Organ Procurement in the Netherlands and Its Impact on Transplant Outcome

**DOI:** 10.3389/ti.2024.12989

**Published:** 2024-06-11

**Authors:** K. A. Chotkan, I. P. J. Alwayn, A. C. Hemke, A. Baranski, W. Nijboer, R. A. Pol, A. E. Braat

**Affiliations:** ^1^ Department of Surgery, Division of Transplantation, Leiden University Medical Center, Leiden, Netherlands; ^2^ Dutch Transplant Foundation, Leiden, Netherlands; ^3^ Transplant Center, Leiden University Medical Center, Leiden, Netherlands; ^4^ Department of Surgery, Division of Transplantation, University Medical Center Groningen, Groningen, Netherlands

**Keywords:** organ donation, organ procurement, surgery, quality and safety, transplant outcomes

## Abstract

In this study, 10 years of procurement quality monitoring data were analyzed to identify potential risk factors associated with procurement-related injury and their association with long-term graft survival. All deceased kidney, liver, and pancreas donors from 2012 to 2022 and their corresponding recipients in the Netherlands were retrospectively included. The incidence of procurement-related injuries and potential risk factors were analyzed. Of all abdominal organs procured, 23% exhibited procurement-related injuries, with a discard rate of 4.0%. In kidneys and livers, 23% of the grafts had procurement-related injury, with 2.5% and 4% of organs with procurement-related injury being discarded, respectively. In pancreas procurement, this was 27%, with a discard rate of 24%. Male donor gender and donor BMI >25 were significant risk factors for procurement-related injury in all three abdominal organs, whereas aberrant vascularization was significant only for the kidney and liver. In the multivariable Cox regression analyses, procurement-related injury was not a significant predictor for graft failure (kidney; HR 0.99, 95% CI 0.75–1.33, *p* = 0.99, liver; HR 0.92, 95% CI 0.66–1.28, *p* = 0.61, pancreas: HR 1.16; 95% CI 0.16–8.68, *p* = 0.88). The findings of this study suggest that transplant surgeons exhibited good decision-making skills in determining the acceptability and repairability of procurement-related injuries.

## Graphical Abstract

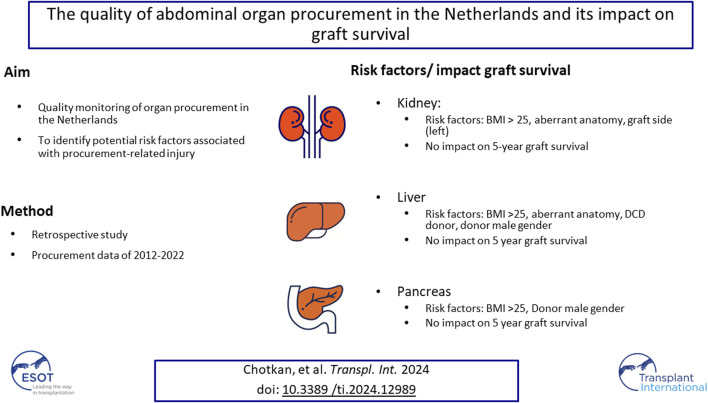

## Introduction

The scarcity of donor organs has created an imbalance between their availability and the growing number of patients on the waiting list. Preventing organ loss due to complications during procurement is paramount, emphasizing the importance of evaluating procurement quality.

In the Netherlands, procurement and transplantation procedures are performed by a dedicated team of surgeons. Over the past decade, the Netherlands has implemented several initiatives to improve procurement quality. In 2010, a national training, certification, and accreditation program was introduced to educate surgeons on abdominal organ procurement procedures [1]. Before this initiative, a data analysis of livers procured in one center in the period 1996–2004 in the Netherlands showed an injury rate of 34% [2]. Subsequently, in 2012, the Quality Form System, a digital scoring system, was implemented to monitor and improve procurement quality and continues to be utilized in the Netherlands. The system involves the completion of a Quality Form for each accepted organ by both the procuring and accepting surgeons after inspection of the organ with data collection by the Dutch Transplantation Foundation. An assessment of procurement-related injuries based on the responses to these Quality Forms was conducted in 2013 [3]. This analysis showed that procurement-related injuries occurred in 25% of procured organs, with a 2% discard rate of organs with procurement related injury. The discard rate due to procurement-related injury was 13% for the pancreas, whereas it was 1% for both kidney and liver. In 23% of cases, there was a discrepancy between the evaluation of the procuring surgeon and transplanting surgeon. As the monitoring system was new, the study only included 1 year of data, resulting in a relatively small sample size of procured organs (270 kidneys, 70 livers, and 28 pancreases) [3].

Monitoring procurement-related injuries is important because of the associated risk of organ discarding. In addition, donor organ procurement-related injuries can be challenging to manage, potentially irreversible, and may lead to diminished graft function post-transplantation. Despite its significance, there is limited literature available on long-term outcomes following procurement-related injuries [2–8]. Ausania et al. conducted a study categorizing surgical injuries in pancreas procurement and found that arterial and parenchymal injuries significantly negatively affect graft survival [5]. This finding underlines the importance of separately evaluating different categories of procurement-related injuries on graft survival. Notably, in some studies, the scoring of injuries by transplanting surgeons was not consistently available. Relying solely on the procuring surgeon to score surgical injuries might introduce a degree of variability and compromise the reliability of the scoring process.

This study aimed to assess the incidence of procurement-related injuries of abdominal organs procured between the period 2012–2022, including more data on procurement quality, and to investigate the effect of procurement-related injury on 5-year graft survival.

## Patients and Methods

### Study Design and Population

This study was a retrospective analysis liver and kidney procured in the Netherlands from March 2012 to December 2022, and all pancreases procured with the intent of whole organ transplantation, between January 2014 and December 2022. The inclusion of pancreases started from 2014 because from that year, information on pancreas acceptance for whole-organ transplantation or islet transplantation was registered. The procurement technique used is described in a National Protocol called *Postmortem* donor organ procurement, made by the Organ advisory committee on organ procurement of the Dutch Transplantation Society [9]. Information regarding the surgical technique is included in the Supplementary Appendix.

### Data Source

The baseline characteristics of the donors were retrieved from the Eurotransplant database. Follow-up data for transplant recipients were sourced from the NOTR (Netherlands Organ Transplant Registry). Consequently, only grafts transplanted in the Netherlands were included in the follow-up analyses. The study protocol was approved by the review board of the NOTR of the Dutch Transplantation Foundation (registration no. 56765) and adhered to the principles outlined in the WMA Declaration of Helsinki and Declaration of Istanbul.

### Quality Form

The Quality Form application is a mandatory system administered by the Dutch Transplantation Foundation. Procuring surgeons were required to complete a form after each procurement procedure. If an organ is transplanted in the Netherlands, the transplanting surgeon reviews the form and confirms agreement or disagreement. The Quality Form encompasses the assessment of organ quality (good, acceptable, and poor), organ injury (yes/no), arterial and venous anatomy (normal/abnormal), and the evaluation of organ injury. In accordance with the classification proposed by de Boer et al., the C1-classification denotes a preventable procurement-related injury with the organ still being transplanted [3]. The C2-classification indicates preventable procurement-related injury resulting in the organ not being transplanted (Table 1). If there was a disagreement of between the form completed by the procuring surgeon and the transplanting surgeon, the responses of the transplanting surgeon were used. In this study, also forms only filled out by the procuring surgeon were used.

**TABLE 1:** Composition of the procurement-related injury classification, C1: organ transplanted, C2: organ not transplanted. Quality Form scoring system according to the system developed by de Boer et al. [2].

Type on procurement-related injury (C)	Example
Arterial	Intima dissection, partial/complete transection, no aortic patch
Venous	Tear, partial/complete transection, no caval patch
Parenchymal	Tear in capsule, parenchymal rupture

### Definitions and Study End Points

The primary outcome measure was the incidence of procurement-related injury. The secondary outcome measures included (death-censored) graft survival, incidence of primary nonfunction (PNF), and delayed graft function (DGF) in kidney transplantation.

For kidney transplant outcomes, DGF was defined as the need for dialysis within the first week after transplantation, while PNF was defined as a non-functioning graft 3 months after transplantation.

For liver transplant outcomes, PNF was defined as the need for re-transplantation or death <7 days after transplantation.

Extraction time of the organ is defined as the time duration between the start cold perfusion of the aorta and the organ’s removal from the donor’s body. The first warm ischemic time was defined as the duration from asystole in the DCD donor until the start of cold perfusion, which is applicable only to DCD donors. Cold ischemic time was defined as the duration from the start of cold perfusion until removal from cold storage or cold machine perfusion at the (receiving) transplant center. The second warm ischemic time (graft anastomosis time) was defined as the time from organ removal from static cold storage or (hypothermic) machine perfusion until reperfusion in the recipient [10]. The Modification of Diet in Renal Disease (MDRD) equation was used to calculate the eGFR in mL/min/1.73 m^2^ [11]. The exclusion of ethnicity was due to its unavailability in the Eurotransplant database.

Aberrant vascular anatomy of the kidney is defined as a kidney graft with multiple renal arteries of renal veins. Aberrant vascular anatomy of the liver and pancreas is defined according to Hiatt’s classification [12].

### Statistical Analysis

Continuous data were presented as mean ± standard deviation (SD). Categorical data were presented as percentages (%) and absolute numbers. The Kolmogorov-Smirnoff test was used to assess whether continuous variables followed a normal distribution. Parametric tests were used to assess the differences between continuous variables. The Chi-square test was used to assess differences between categorical data. Statistical significance was set at *p* < 0.05.

To assess the potential associations between procurement-related injury of an organ and other variables, a binary logistic regression analysis (procurement-related injury versus no injury) was performed. Initially, each variable was analyzed using a univariable logistic regression model, followed by a multivariate model.

For follow up analysis only the ‘C1’ category organs (procurement-related injury, organ transplanted) were used. Univariate and multivariable (stepwise) binary logistic regression analyses were employed to determine associations between donor, recipient, and procedural characteristics and DGF in kidney transplant recipients. The results are presented as odds ratios (OR) with corresponding confidence intervals (CI) and *p*-values; Kaplan-Meier survival curves were used to assess death-censored graft survival, and the log-rank test was used to determine differences between the no procurement-related injury and procurement-related injury groups. Recipients who died with a functioning graft were censored, whereas recipients who died due to graft failure were not censored. Univariable and multivariable (stepwise) Cox regression analyses were performed to identify associations between donor, recipient, procedural characteristics and death-censored kidney- and liver graft survival. Results were presented as hazard ratios (HR) with corresponding confidence intervals (CI) and *p*-values. Linear mixed models were used to evaluate the mean change in kidney function (expressed as eGFR) over the first 6 years post-transplantation. To assess the longitudinal effect of kidneys with no procurement-related injury versus kidneys with procurement-related injury on eGFR, we defined procurement-related injury, post-transplant time in years, and the interaction between procurement-related injury and post-transplant time as fixed effects.

For statistical analyses, IBM SPSS Statistics for Windows was used (IBM Corp. Released 2022. Version 29.0).

## Results

### Kidney

**FIGURE 1:**
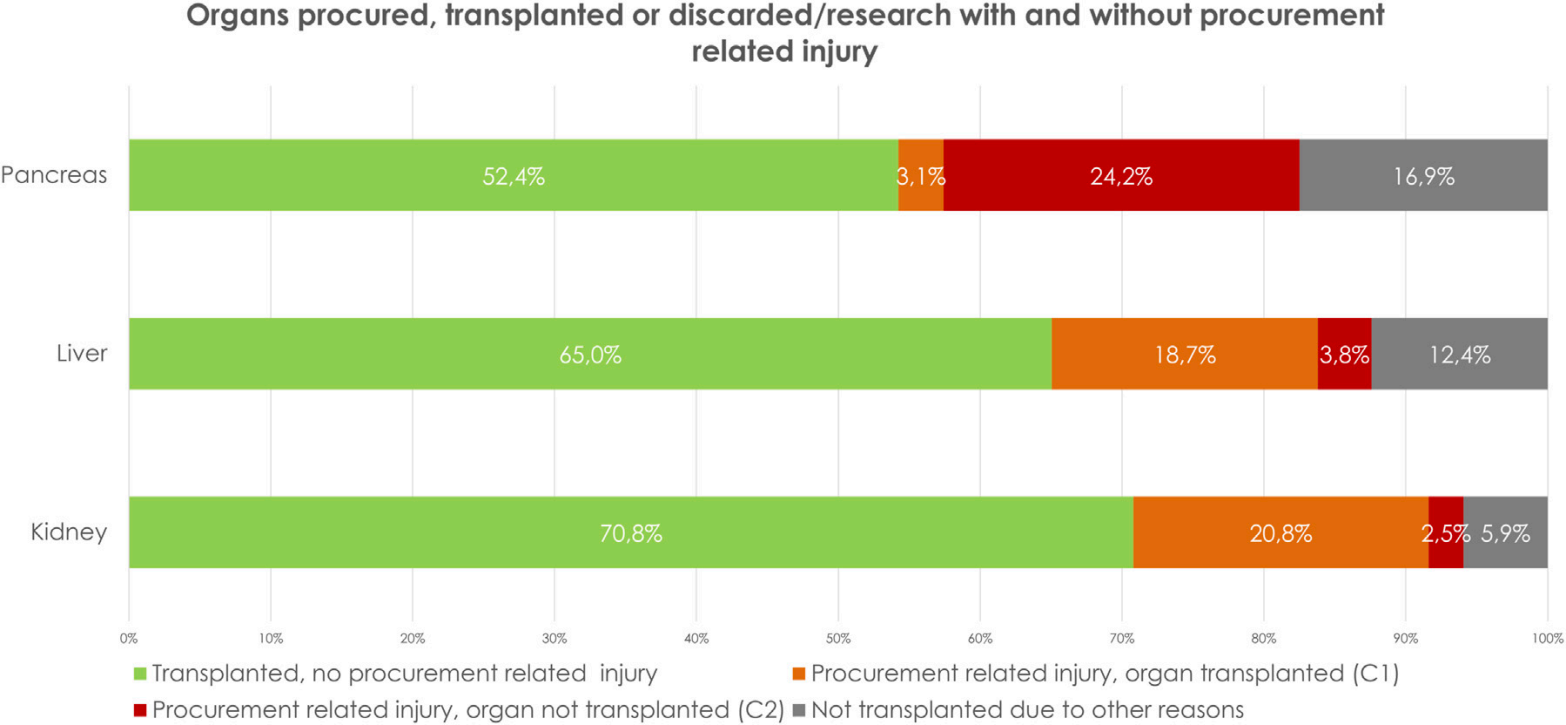
Organs procured, transplanted or discarded/research with and without procurement related injury.

Between March 1st^,^ 2012, and December 31st^,^ 2022, 5,495 kidneys were procured, and 5,034 kidneys were transplanted. In total, 461 (8.5%) of the procured kidney grafts were not transplanted (Figure 1; Table 2). Of the procured kidneys, 73% (n = 4,003) had one renal artery, 21% (n = 1,176) had two renal arteries and 3% (n = 171) had three renal arteries and in 2% (n = 118) this was not reported. Almost 91% (n = 4,987) had one renal vein, 7% (n = 382) had two renal veins and 1% (n = 31) three renal veins, in 2% (n = 92) the number of veins was not reported.

**TABLE 2:** **A:** Number of reported, procured, and transplanted organs. **B:** Procurement related injury per organ as percentage of the total number of organ type procured.

	Kidney	Liver	Pancreas	Total
**A**				
Total number of organs procured with intend of transplantation	5,495 (100%)	2093 (100%)	456 (100%)	8,044
Total number of transplanted organs	5,034 (91.5%)	1753 (83.8%)	253 (55%)	7,040
**B**				
Procurement related injury, organ transplanted (C1)	20.8% (n = 1,144/5,495)	18.7% (n = 392/2093)	3.1% (n = 14/456)	19.3% (1,550/8,044)
Procurement related injury, organ not transplanted (C2)	2.5% (n = 135/5,495)	3.8% (n = 79/2093)	24.2% (n = 110/456)	4.0% (n = 324/8,044)
Total percentage of injury	23.3% (n = 1,279/5,495)	22.5% (n = 471/2093)	27.2% (n = 124/456)	23.3% (n = 1874/8,044)

In 1,279 grafts (23.3%) there was procurement related injury (C1+C2), of which 1,144 grafts were classified as C1 (repaired and transplanted) and 135 (2.5%) as C2 (not transplanted) (Figure 1; Table 2). Parenchymal injury was the most frequent injury type (Table 3). Stratifying by donor type, DCD donors had a significantly higher percentage of procurement-related injuries (C1: 20.7% vs. 20.9%, C2: 1.6% vs. 3.1%, *p* < 0.01) (Table 4). Additionally, a higher incidence of procurement-related injury was observed in left kidney grafts (left grafts; C1: 27%, C2: 3%, right grafts; C1: 15%, C2: 2%, *p* < 0.01). Venous injury was more frequent in left kidney grafts (58% vs. 42%,*p* < 0.01), whereas arterial injury was more frequent in right kidney grafts (43% vs. 57%, *p* < 0.01).

**TABLE 3:** Type of procurement related injury, kidney (percentages as total of the procured kidneys with procurement related injury) and liver (percentages as total of the procured livers with procurement related injury).

	C1	C2	Total
Kidney			
Arterial	n = 110 (8.6%)	n = 18 (1.4%)	128 (10.0%)
Venous	n = 50 (3.9%)	n = 9 (0.7%)	59 (4.6%)
Parenchymal related	n = 216 (17.0%)	n = 26 (2.0%)	242 (19%)
Not classified	n = 768 (60%)	n = 82 (6.4%)	850 (66.4%)
Total	1,144 (89%)	135 (11%)	1,279 (100%)
Liver			
Arterial	n = 125 (26.5%)	n = 26 (5.5%)	n = 151 (32%)
Venous	n = 38 (8.1%)	n = 0 (0%)	n = 38 (8.1%)
Parenchymal related	n = 199 (42.2%)	n = 42 (8.9%)	n = 241 (51.1%)
Not classified	n = 30 (6.4%)	n = 11 (2.3%)	n = 41 (8.7%)
Total	n = 392 (83.2%)	n = 79 (16.8%)	n = 471 (100%)

**TABLE 4:** Procurement related damage per organ type as percentage of the total number of procured organ type, stratified by type of donor.

	DBD			DCD			
	C0	C1	C2	C0	C1	C2	
**Kidney**	77.7% (1805/2,324)	20.7% (n = 482/2,324)	1.6% (n = 37/2,324)	76.0% 2,411/3,171	20.9% (n = 662/3,171)	3.1% (n = 98/3,171)	**p=<0.01** ^a^
**Liver**	79.7% (952/1,194)	18.6% (n = 222/1,194)	1.7% (n = 20/1,194)	74.5% (n = 670/899)	18.9% (n = 170/899)	6.6% (n = 59/899)	**p=<0.01** ^a^
**Pancreas**	72.4% (n = 192/265)	3.8% (n = 10/265)	23.8% (n = 63/265)	73.3% (n = 140/191)	2.1% (n = 4/191)	24.6% (n = 47/191)	*p* = 0.58^a^

DBD, donation after brain death; DCD, donation after circulatory death.

^a^ A Chi-square test (and Fisher extact for the pancreases) was used to investigate whether the incidence of C1 and C2 was different between donor type. Significant differences in bold.

Bold values indicate statistical siginificance of *P* values.

Comparing extraction time between C1-, C2- and no procurement related damage-grafts, showed no significant differences in DBD donors. In DCD donors, the extraction time was significantly longer in procurement related damaged grafts compared to grafts with no procurement related damage (C1 0:56 ± 0:32, C2 0:55 ± 0:34, no procurement related damage 0:52 ± 0:27, *p* = 0.02) (Table 5).

**TABLE 5:** Extraction time, stratified per organ, type of donor and procurement related injury.

	C1	C2	No procurement related damage		Missing data (%)
DBD, Kidney	1:00 ± 0:30	1:04 ± 0:29	0:58 ± 0:26	*p* = 0.26	4
DCD, Kidney	0:56 ± 0:32	0:55 ± 0:34	0:52 ± 0:27	**p = 0.02**	
DBD, Liver	0:47 ± 0:21	0:54 ± 0:27	0:45 ± 0:20	*p* = 0.13	5
DCD, Liver	0:51 ± 0:26	0:46 ± 0:16	0:49 ± 0:22	*p* = 0.41	
DBD, Pancreas	0:58 ± 0:22	0:57 ± 0:26	0:55 ± 0:24	*p* = 0.62	14
DCD, Pancreas	1:17 ± 0:43	1:03 ± 0:39	0:59 ± 0:30	*p* = 0.09	

Bold values indicate statistical siginificance of *P* values.

#### Risk Factors Associated With Injury

In univariable logistic regression analysis, donor male gender, left kidney graft, graft with multiple arteries, and donor BMI >25 were all found to be significantly associated with a higher risk of procurement-related injury (C1+C2) The risk of procurement related injury increased when the number of renal arteries increased (Table 6). In multivariable logistic regression analysis (including donor -gender, donor type, age, BMI, left or right kidney, number of arteries, and number of veins), donor BMI >25, left kidney graft, and a graft with multiple arteries remained significantly associated with a higher risk of procurement-related injury (C1+C2) (Table 6).

**TABLE 6:** Odds ratios of risk factors for procurement related injury (C1+C2), kidney.

	Univariable		Multivariable^a^	
Donor gender				
- Female	1.00	***p*<0.01**	1.00	*p* = 0.08
- Male	1.20 [1.05–1.39]		1.13 [0.99–1.29]	
Donor type - DBD - DCD	1.00 1.01 [0.99–1.24]	*p* = 0.16	1.00 1.05 [0.93–1.20]	*p* = 0.44
Donor age - 0–15 years - 16–25 years - 26–35 years - 36–45 years - 46–55 years - 56–65 years - 66–75 years - >75 years	0.80 [0.48–1.33] 1.00 1.10 [0.78–1.56] 1.08 [0.78–1.50] 1.16 [0.88–1.54] 1.20 [0.91–1.58] 1.20 [0.90–1.59] 0.89 [0.50–1.58]	*p* = 0.53	0.94 [0.53–1.67] 1.00 1.05 [0.73–1.51] 1.08 [0.77–1.51] 1.16 [0.86–1.55] 1.14 [0.85–1.52] 1.19 [0.88–1.59] 0.93 [0.52–1.68]	*p* = 0.90
Donor BMI (kg/m^2^) - <18.5 - 18,5–25 - 25–30 - 30–35 - 35–40 - >40	0.87 [0.60–1.24] 1.00 1.32 [1.15–1.52] 1.48 [1.20–1.84] 1.42 [1.02–1.97] 1.12 [0.64–1.97]	***p*<0.01**	0.93 [0.62–1.41] 1.00 1.26 [1.09–1.47] 1.44 [1.15–1.81] 1.38 [0.98–1.93] 1.08 [0.61–1.95]	***p*<0.01**
Graft side - Right kidney - Left kidney	1.00 2.13 [1.89–2.44]	***p*<0.01**	1.00 2.16 [1.89–2.47]	***p*<0.01**
Number of arteries - One - Two - Three - Four	1.00 1.41 [1.22–1.64] 1.74 [1.25–2.41] 5.70 [2.46–13.20]	***p*<0.01**	1.00 1.40 [1.20–1.63] 1.75 [1.25–2.45] 5.26 [2.22–12.46]	***p*<0.01**
Number of veins - One - Two - Three	1.00 0.85 [0.66–1.09] 0.62 [0.23 = 1.60]	*p* = 0.20	1.00 1.04 [0.79–1.35] 0.90 [0.34–2.38]	*p* = 0.79

^a^ In the multivariable analysis donor-gender, -age, -type, -BMI, the graft side, number of arteries and veins of the graft are all added at once in the same model.

Bold values indicate statistical siginificance of *P* values.

#### Follow up of Kidneys Transplant Recipients With Procurement-Related Injury (C1)

A total of 5,034 kidneys were transplanted, of which 4,094 were transplanted in the Netherlands. The follow-up data for 4% was missing (n = 160), resulting in the inclusion of 3,934 kidney recipients in the follow up analyses. In 83% the Quality Form was completed by both the procurement surgeon and the transplant surgeons. In 16% the transplant surgeon disagreed with the procuring surgeon on at least one subject.

The characteristics of the kidney donors, recipients and the procedure are summarized in Supplementary Table S1, stratified by the absence (C0) or presence of procurement-related injury (C1). A significant difference was observed in donor BMI and donor gender. In total, 23% of the recipients (n = 909) received a kidney with (repaired) procurement-related injury. Most baseline characteristics were not significantly different, except that there were significantly more left kidney grafts in the C1 group (65% vs. 46%, *p* < 0.01). Additionally, more grafts in the C1 group had multiple arteries (23% vs. 33%, *p* < 0.01).

#### Short Term Transplant Outcome

DGF was observed in 35% (n = 1,376) of recipients, while PNF occurred in three percent (n = 120). Eight percent of the information on graft function in the first week after transplantation was missing. When comparing the incidence of immediate graft function, DGF, and PNF separately for recipients of DBD and DCD donors, no significant differences were observed between the C0 and C1 groups (Table 7). Comparing the incidence of immediate graft function, DGF and PNF separate per type of damage group, arterial, venous, and parenchymal related damage versus no procurement related damage, the incidence of PNF was higher in grafts from DBD donors with venous damage compared to grafts from DBD with no procurement related damage (14% versus 2.5%, *p* < 0.01). The incidence of DGF was significantly higher in grafts from DBD and DCD donors with parenchymal damage (39% versus 21% in kidney grafts from DBD donors, 56% versus 46% in kidney grafts from DCD donors) (Table 7).

**TABLE 7:** Graft function in kidney recipients, stratified by donor type, procurement related damage (no/yes: C1), and type of damage.

		Immediate graft function	Delayed graft function	Primary non function	
**DBD**					
No Procurement related damage		73% (n = 799)	21% (n = 233)	2.5% (n = 27)	*p* = 0.72
Procurement related damage (C1)		71% (n = 226)	25%(n = 78)	2.5% (n = 8)	
Type of damage					
	Arterial damage (vs. no damage)	68% (n = 28)	27%(n = 11)	5% (n = 2)	*p* = 0.44
	Venous damage (vs. no damage)	64% (n = 9)	7% (n = 1)	14% (n = 2)	***p*<0.01**
	Parenchymal damage (vs. no damage)	61% (n = 30)	39% (n = 19)	0% (n = 0)	**p = 0.02**
**DCD**					
No Procurement related damage		48% (n = 826)	46% (n = 800)	4% (n = 72)	*p* = 0.11
Procurement related damage (C1)		47% (n = 246)	50% (n = 265)	2.4% (n = 13)	
Type of damage					
	Arterial damage (vs. no damage)	46% (n = 22)	48% (n = 23)	4% (n = 2)	*p* = 0.99
	Venous damage (vs. no damage)	41% (n = 11)	59% (n = 16)	0% (n = 0)	*p* = 0.43
	Parenchymal damage (vs. no damage)	43% (n = 50)	56% (n = 65)	0% (n = 0)	**p = 0.04**

Values are presented as percentage. DBD, Donation after Brain death**;** DCD, donation after circulatory death.

^a^ A Chi-square test was used to investigate the difference in incidence in immediate graft function, delayed graft function and primary non function between the groups.

Bold values indicate statistical siginificance of *P* values.

Univariate logistic regression demonstrated that procurement-related injury did not increase the risk of developing DGF (OR, 1.14; 95% CI 0.98–1.34, *p* = 0.10) (Table 8). In multivariable logistic regression analyses, this was confirmed after adjustment for potential confounding factors (Table 8, models 1–3). Donor age, body mass index, male gender, history of hypertension, cause of death, type of donor (DCD), recipient age, history of diabetes and cardiac disease, cold ischemic time, and preservation method (cold storage) were associated with a higher risk of developing DGF based on multivariate analyses (Supplementary Table S2).

**TABLE 8:** Uni- and multivariable logistic regression analysis and Cox regression analysis evaluating the association between procurement-related injury, correcting for donor, procedural and recipient characteristics with the risk of delayed graft function and (death censored) graft failure in the kidney recipient. Results of the full model are listed in Supplementary Table S2.

	DGF OR [95% CI]		Graft failure HR [95% CI]	
Univariable	1.14 [0.98–1.34]	*p* = 0.10	0.94 [0.77–1.14]	*p* = 0.54
Model 1	1.10 [0.93–1.30]	*p* = 0.27	0.94 [0.78–1.15]	*p* = 0.58
Model 2	1.41 [0.8–1.27]	*p* = 0.97	0.94 [0.71–1.25]	*p* = 0.68
Model 3	1.02 [0.81–1.31]	*p* = 0.85	0.99 [0.75–1.33]	*p* = 0.99

Model 1: Procurement-related injury + donor age + donor BMI + donor gender + donor history of diabetes + donor history of hypertension + donor type + donor cause of death.

Model 2: Model 1 + first warm ischemia time + second warm ischemia time + cold ischemia time + multiple arteries + multiple veins + kidney site + machine perfusion.

Model 3: Model 2 + recipient age + recipient BMI + recipient gender + recipient diabetes + recipient cardiac disease + primary disease.

Univariable = procurement-related injury, C1 only.

#### Long Term Transplant Outcome

**FIGURE 2:**
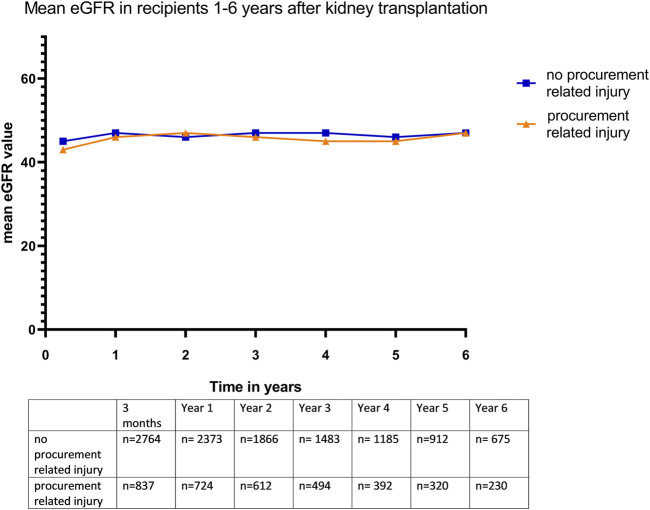
Mean eGFR (in mL/min/1.73m^2^) in kidney recipients 3 months −6 years after transplantation.

In a linear mixed model using eGFR as the dependent variable, there was no significant difference in the mean eGFR over time between the C0 and C1 groups at 3 months and 1–6 years post transplantation (*p* = 0.77) (Figure 2).

**FIGURE 3:**
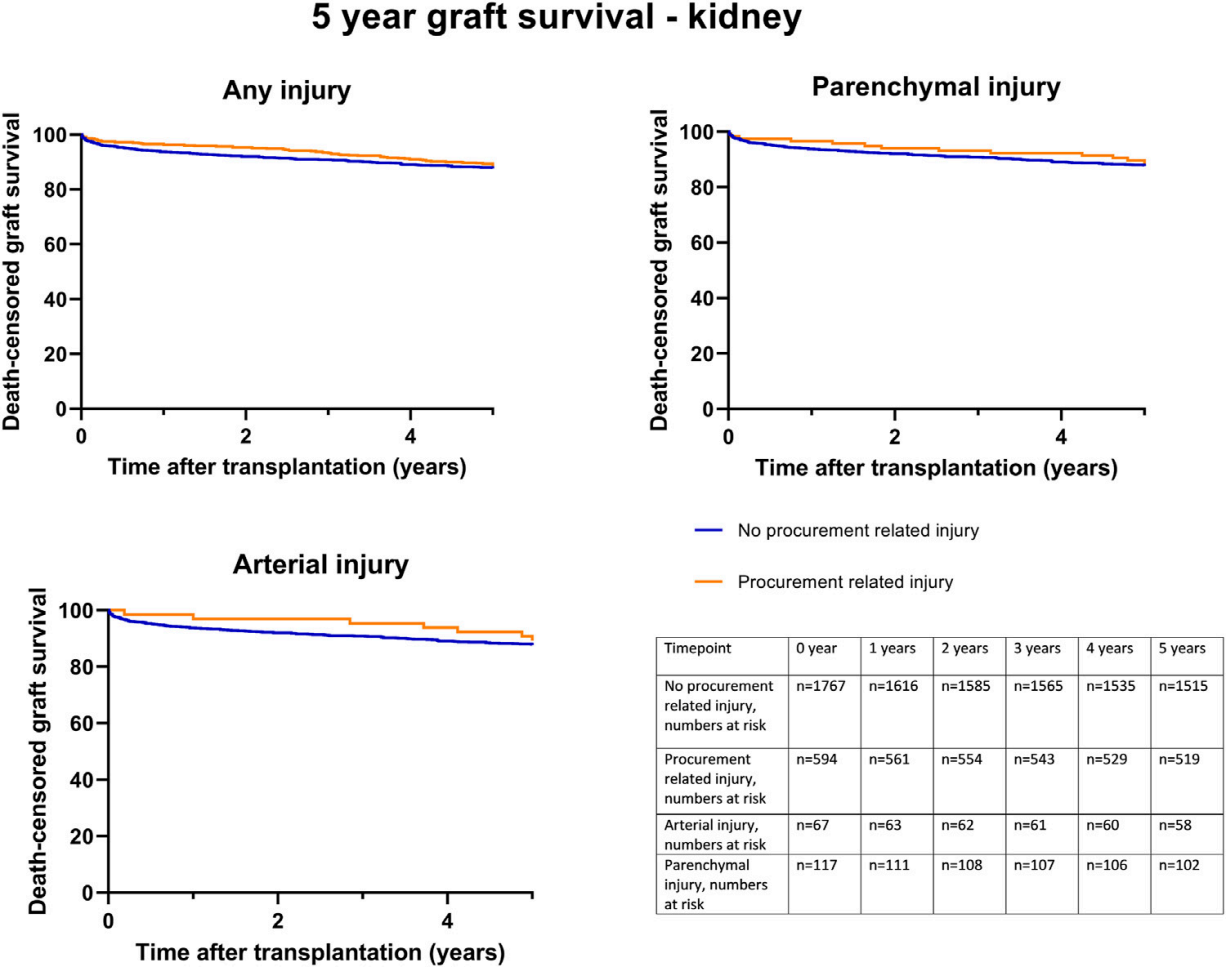
Death-censored graft survival until 5 years post kidney transplantation, according to procurement related injury; any C1 injury, (Log rank test *p* = 0.44), parenchymal injury (Log rank test *p* = 0.59) and arterial injury (Log rank test *p* = 0.78).

Kaplan-Meier survival analysis showed no significant differences in death-censored graft survival 5 years post transplantation between the C0 and C1 groups (log-rank test, *p* = 0.44) (Figure 3). A separate Kaplan-Meier survival analysis was performed for parenchymal and arterial injuries, which also showed no significant differences.

Univariable death-censored Cox regression analyses demonstrated that procurement-related injury did not increase the hazard rate of graft failure (HR 0.94; 95% CI 0.77–1.14, *p* = 0.54) (Table 8). This finding was further confirmed by multivariable Cox regression analysis adjusted for potential confounding factors (Table 8, model 1–3). Donor age, recipient age, history of cardiac disease, and cold ischemic time were identified as the significant factors associated with graft failure (Supplementary Table S2).

### Liver

Between March 1st^,^ 2012, and December 31st^,^ 2022, 2093, livers were procured and 1753 were transplanted. In total, 340 (16.2%) of the procured liver grafts were not transplanted. 112 grafts were procured *en-bloc* with the pancreas.

Of the procured organs, 69.5% (n = 1,455) had a normal vascular anatomy. 14% (n = 292) had replaced or accessory left hepatic artery (Type II), 7% (n = 145) a replaced or accessory right hepatic artery (Type III), 3% (n = 61) a replaced or accessory right hepatic artery + replaced or accessory left hepatic artery (Type IV), 1% (n = 13) had the common hepatic artery arise from the superior mesenteric artery (Type V) and 0.1% (n = 2) had the common hepatic artery arise from the aorta (Type VI). In 6% (n = 125) no further classification of the aberrant anatomy was available. In 471 grafts (22.5%) there was procurement related injury (C1+C2), of which 392 grafts were classified as C1 (repaired and transplanted) and 79 (3.8%) as C2 (not transplanted) (Figure 1; Table 2). Stratifying for the type of injury, parenchymal injury emerged as the most frequent type of injury (Tables 2, 3). Stratifying donor-type DCD donors had a significantly higher percentage of procurement-related injury for both C1 and C2 (C1: 18.9. % vs. 18.6%, C2; 6.6% vs. 1.7%, *p*=<0.01) (Table 4). Comparing extraction time between C1-, C2- and no procurement related damage-grafts, showed no significant differences (Table 5).

#### Risk Factors Associated With Injury

In univariate logistic regression analysis, donor-male gender, BMI> 25, DCD type of donor, and aberrant vascular anatomy were all significantly associated with a higher risk of procurement-related injury (C1+C2) (Table 9). Especially type III and type VI of aberrant vascular anatomy were associated with a higher risk of procurement related injury. Multivariable logistic regression analysis (including donor, type, age, BMI, and aberrant vascular anatomy) confirmed these associations (Table 9).

**TABLE 9:** Odds ratios of risk factors for procurement related injury (C1+C2), liver.

	Univariable		Multivariable	
Donor gender - Female - Male	1.00 1.33 [1.09–1.64]	**p = 0.03**	1.00 1.24 [1.01–1.54]	**p = 0.05**
Donor type - DBD - DCD	1.00 1.34 [1.09–1.65]	***p*<0.01**	1.00 1.31 [1.05–1.52]	***p*<0.01**
Donor age - 0–15 years - 16–25 years - 26–35 years - 36–45 years - 46–55 years - 56–65 years - 66–75 years > 75 years	0.74 [0.34–1.62] 1.00 1.06 [0.62–1.79] 1.10 [0.68–1.79] 0.98 [0.64–1.50] 1.05 [0.69–1.60] 0.91 [0.58–1.43] 0.70 [0.32–1.51]	*p* = 0.88	0.78 [0.33–1.83] 1.00 1.02 [0.60–1.75] 1.08 [0.66–1.76] 0.94 [0.61–1.44] 0.99 [0.64–1.52] 0.93 [0.58–1.47] 0.76 [0.34–1.68]	*p* = 0.98
Donor BMI (kg/m^2^) - <18.5 - 18,5–25 - 25–30 - 30–35 - 35–40 - >40	1.02 [0.58–1.78] 1.00 1.45 [1.15–1.83] 1.54 [1.08–2.20] 1.41 [0.77–2.59] 3.15 [1.25–7.97]	***p*<0.01**	1.12 [0.61–2.08] 1.00 1.39 [1.10–1.77] 1.38 [0.96–1.99] 1.42 [0.77–2.63] 3.16 [1.23–8.13]	**p = 0.03**
Anatomy vascularization^b^ - Normal - Type II - Type III - Type IV - Type V - Type VI - Not classified	1.00 0.99 [0.73–1.36] 2.15 [1.49–3.10] 1.15 [0.62–2.11] 1.15 [0.32–4.22] 3.85 [0.24–61.7] 1.88 [1.26–2.79]	***p*<0.01**	1.00 1.01 [0.74–1.34] 2.13 [1.47–3.08] 1.12 [0.60–2.07] 1.04 [0.28–3.87] 3.98 [0.24–65.11] 1.82 [1.22–2.71]	***p*<0.01**

^a^ In the multivariable analysis donor-gender, -type, -age -BMI, and normal/abnormal anatomy regarding vascularization are all added at once in the same model.

^b^ According to Hiat’s classification: Type I: normal anatomy; Type II: replaced or accessory left hepatic artery; Type III: replaced or accessory right hepatic artery; Type IV: replaced or accessory right hepatic artery + replaced or accessory left hepatic artery; Type V: common hepatic artery from the superior mesenteric artery; Type VI: common hepatic artery from the aorta.

Bold values indicate statistical siginificance of *P* values.

#### Follow up of Liver Transplant Recipients With Procurement-Related Injury (C1)

In total, 1753 livers were transplanted, with 1,317 whole livers transplanted in the Netherlands, which formed the basis for the follow-up analyses. In 86% (n = 1,136) the Quality Form was completed by both the procurement surgeon and the transplant surgeon. In 30% the transplant surgeon disagreed with the procuring surgeon on at least one subject. In these cases, the response of the transplant surgeon was used.

The characteristics of liver donors and their recipients are outlined in Supplementary Table S3, stratified by the presence or absence of procurement-related injury (C0 vs. C1). There was a significant difference in the BMI between the groups (*p*=<0.01). In total, 23% of the recipients (n = 306) received a liver with (repaired) procurement-related injury (C1). No significant differences were observed in the baseline characteristics between the two groups.

#### Transplant Outcome

Twenty-five recipients (1.9%) had PNF. The incidence of PNF was not significantly different between the C0 and C1 groups of recipients (C0: 6%, n = 18 versus C1: 9%, n = 7, *p* = 0.15). In addition, the incidence of graft related injuries, (anastomotic biliary complications, hepatic vein thrombosis, and arterial thrombosis taken together), other reasons for graft failure (i.e., recurrence of disease, malignancy *de novo*, rejection, bacterial infection) and no graft failure were compared between grafts with no injury and C1-injury. This showed no significant difference in incidence.

**FIGURE 4:**
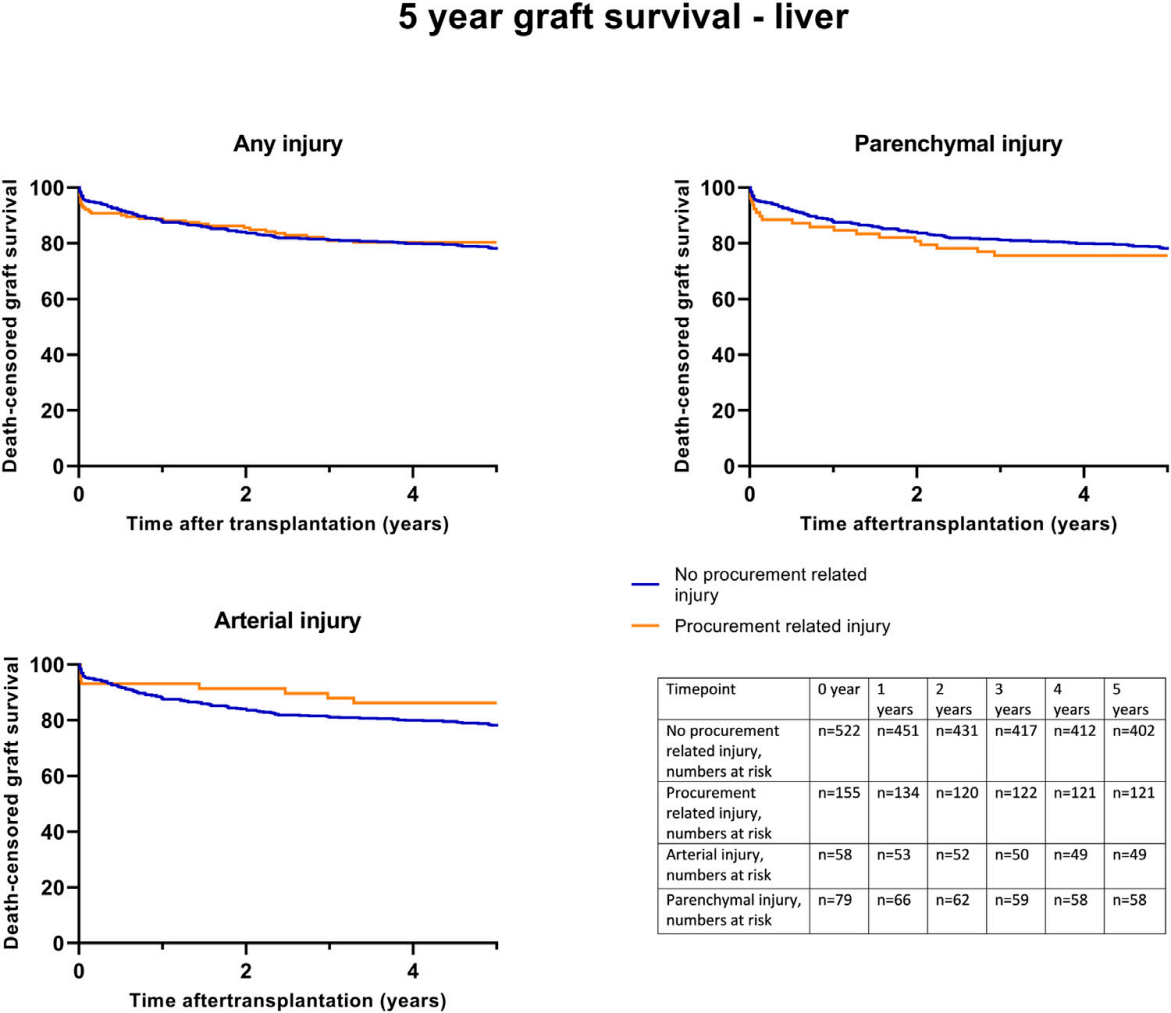
Death-censored graft survival until 5 years post liver transplantation, according to procurement related injury; any C1 injury, (Log rank test *p* = 0.74), parenchymal injury (Log rank test *p* = 0.30) and arterial injury (Log rank test *p* = 0.45).

Kaplan-Meier survival analysis showed no significant differences in death-censored graft survival 5 years post-transplantation between the C0 and C1 groups (log-rank test *p* = 0.74) (Figure 4). Further Kaplan-Meier survival analysis, specifically for parenchymal and arterial injuries, also demonstrated no significant differences.

Univariable death-censored Cox regression analyses indicated that procurement-related injury did not increase the hazard rate for graft failure (0.91; 95% CI [0.67–1.21], *p* = 0.51). This finding was confirmed by multivariable Cox-regression analysis adjusted for potential confounding factors (Table 10, model 1–3). Notably, donor age, type (DCD), recipient age, and primary disease were identified as significant factors for graft failure (Supplementary Table S4).

**TABLE 10:** Uni- and multivariable Cox regression analysis evaluating the association between procurement-related injury, correcting for donor, procedural and recipient characteristics with the risk of (death censored) graft failure in the liver recipient. Results of the full model are listed in Supplementary Table S3.

	Graft failure HR [95% CI]	
Univariable (procurement related injury, C1)	0.89 [0.66–1.20]	*p* = 0.46
Model 1	0.90 [0.65–1.25]	*p* = 0.90
Model 2	0.90 [0.65–1.25]	*p* = 0.51
Model 3	0.92 [0.66–1.28]	*p* = 0.61

Model 1: Procurement-related injury + donor age + donor BMI + donor gender + donor history of diabetes + donor history of hypertension + donor type + donor cause of death.

Model 2: Model 1 + first warm ischemia time + second warm ischemia time + cold ischemia time + aberrant vascular anatomy.

Model 3: Model 2 + recipient age + recipient BMI + recipient gender + primary disease.

### Pancreas

Between January 1st^,^ 2014, and December 31st^,^ 2022, 456 pancreases were procured for whole organ transplantation, 253 pancreases were transplanted as whole organs, and 16 were eventually used for islet transplantation. In total, 187 (41%) pancreases were not used of transplantation, of which 24% (n = 110) had procurement-related injury (C2) (Figure 1; Table 2). Eight of the grafts C2 grafts were used for islet transplantation and 28 were used for research.

Three percent of pancreases (13/456 pancreases procured) were classified as ‘C1.’ After stratification by donor type, no significant differences were found in the percentages of ‘C1’ and ‘C2’ between DBD and DCD donors (Table 4).

Comparing extraction time between C1-C2- and no procurement related damage-grafts, showed no significant differences (Table 4).

#### Risk Factors Associated With Injury

Univariate binary logistic regression analyses showed that a donor BMI >25 was significantly associated with a higher risk of procurement-related injury (Table 11).

**TABLE 11:** Odds ratios of risk factors for procurement related injury (C1+C2), pancreas.

	Univariable		Multivariable	
Donor gender - Female - Male	1.00 1.55 [0.99–2.42]	*p* = 0.06	1.00 1.71 [1.06–2.75]	**p = 0.03**
Donor type - DBD - DCD	1.00 1.10 [0.70–1.72]	*p* = 0.69	1.00 1.13 [0.69–1.84]	*p* = 0.64
Donor age - 0–15 years - 16–25 years - 26–35 years - 36–45 years - 46–55 years - 56–65 years - 66–75 years	0.66 [0.23–1.95] 1.00 0.84 [0.39–1.77] 1.24 [0.63–2.45] 1.66 [0.89–3.10] 0.46 [0.12–1.70] 1.75 [0.39–7.90]	*p* = 0.21	0.80 [0.27–2.41] 1.00 0.88 [0.41–1.87] 1.36 [0.68–2.73] 1.89 [0.97–3.69] 0.41 [0.11–1.57] 1.65 [0.35–7.78]	*p* = 0.13
Donor BMI (kg/m^2^)^a^ - <25 - >25	1.00 1.73 [1.11–2.71]	**p = 0.02**	1.00 1.65 [0.35–7.78]	**p = 0.04**

^a^ Because 55% of the donors had a BMI, of 18,5%–25% and 34% of the donors had a BMI, of 25–30, and the number of donors in the other categories (<18,5, 30–35, >40) were small, this division was chosen.

Bold values indicate statistical siginificance of *P* values.

In the multivariable binary logistic regression analysis (including donor age, gender, BMI, and type), both BMI >25 and male gender emerged as significant risk factors for procurement-related injury (Table 11).

#### Follow up of Pancreas Transplant Recipients With Procurement-Related Injury (C1)

A total of 209 pancreases were transplanted into the Netherlands. In 86% of procured grafts, the Quality Form was completed, by both procuring and transplanting surgeons. In 14% disagreements arose regarding at least one subject.

Follow up data of 193 (96%) of the pancreas recipients were accessible in the database. Of the 13 pancreases transplanted with procurement-related injury, ten grafts were transplanted in the Netherlands with available follow-up data. The characteristics of pancreas donors and their recipients are outlined in Supplementary Table S5, stratified by the presence or absence of procurement-related injury (C0 vs. C1). There were no significant differences in baseline characteristics. Kaplan-Meier survival analysis showed no significant differences in death-censored graft survival 5 years post-transplantation between the C0 and C1 groups (log-rank test *p* = 0.86) (Supplementary Figure S1).

Univariable death-censored Cox regression analysis indicated that procurement-related injury did not increase the hazard rate for graft failure (HR 1.16; 95% CI 0.16–8.68, *p* = 0.88). Multivariable analysis was not performed due to small number of cases.

## Discussion

This national study is an extension of the study from 2017 by de Boer et al., including data from 10 years of procurement quality monitoring in the Netherlands. From all organs procured between March 2012-December 2022 (kidney + liver) and January 2014- December 2022 (pancreas); 23% (1874/8,044) had procurement-related injury (C1+C2). Of the injured organs, 4% (324/1874 organs, C2) were not transplanted. Remarkably, the rate of procurement-related injury for pancreatic grafts was notably higher at 27.2%, compared to kidney (23.3%) and liver (22.5%) grafts. Importantly, procurement-related injury did not influence death-censored 5-year graft survival.

In kidney and liver grafts, the ratios of C1-type and C2-type injuries were comparable (kidney C1: 20.8%, C2 2.5%, liver C1: 18.7%, C2: 3.8%), while in pancreatic grafts, the ratio was reversed (C1: 3.1%, C2: 24.2%), suggesting that injured pancreases are more often discarded for transplantation compared to kidney and liver grafts (Table 2; Figure 1). This tendency may stem from transplant surgeons’ reluctance to use an injured pancreatic graft for whole-organ transplantation.

The percentage of procurement-related injury was significantly higher in the DCD procedures than in the DBD procedures for kidney and liver grafts (Table 4). Potential contributing factors include the absence of circulation in DCD donation, making it more challenging to inspect vascular anatomy. In addition, time pressure to minimize warm ischemia and extraction times in DCD procedures may have been a factor, as prolonged nephrectomy and hepatectomy times are associated with worse outcomes after transplantation [13–15]. However, in multivariable analyses, DCD was found to be a significant risk factor for procurement-related injury of the liver, but not for the kidney or pancreas (Tables 5, 9, 11). In DCD liver donation, the entire liver dissection occurs after aortic cross-clamping, whereas in DBD donors, preparatory dissection is performed before the start of aortic cold flushing [16]. In kidney and pancreas procurement, there is less or no preparatory dissection, even in DBD procedures, which could explain why DCD donation was not a significant risk factor for kidney and pancreas procurement.

Higher BMI and male gender of the donor are risk factors for procurement-related injury in kidney, liver, and pancreas procurement, which is supported by other publications [3, 17]. A possible explanation for this association could be variations in fat distribution between genders. Men tend to store body fat in the abdominal (visceral) region, whereas women have a higher proportion of body fat in the gluteal-femoral region [18]. Increased visceral abdominal fat may contribute to the complexity of the procurement procedure. Potential strategies to minimize the risk of procurement related injury in high BMI patients could be to implement an upper limit for accepting donors with a BMI above 40. However, such a measure might have undesirable consequences due to the impact on donor numbers, particularly given the organs shortage.

Left kidney grafts carry a significantly higher risk of procurement-related injury (OR 2.13, 95% CI 1.89–2.44) according to our study. The percentage of left kidney grafts was higher in grafts with procurement-related injury than in those with no procurement-related injury. Venous-related injuries were more frequent in the left kidney than in the right. A possible explanation for this could be the position of the left renal vein on the ventral side of the aorta, enlarging the chance on procurement-related injury of the vein during dissection of the aorta. This result contrasts with a prior study of Taber-Hight et al., which found the right kidney to be the most likely injured organ during procurement, for which we have no clear explanation [19].

We found that kidney and liver grafts with aberrant vascular anatomy (having more than one renal artery in case of kidney procurement, and aberrant anatomy of the liver vascularization according to Hiatt’s classification) were injured more frequently [12]. Knowledge of this anatomy, through the availability of a preoperative contrast-enhanced CT scan, before procurement could aid in preventing procurement-related arterial injuries [20, 21]. In the Netherlands, a contrast-enhanced (abdominal) CT scan has been performed for every DBD and DCD donor since 2023; however, the results of these policy changes on procurement-related injuries are still in progress. Specific risks related to vascular anatomy in pancreas procurement were not analyzed in this study because of the relatively small number of pancreases with this type of anatomy (the hepatic artery arising from the SMA was only reported in 13 donors). Ausenia et al., however, identified the hepatic artery arising from the SMA as a significant risk factor for procurement-related injury in pancreas procurement [5].

This study had a few limitations that need to be acknowledged. First, only grafts transplanted in the Netherlands had a quality form filled out by the transplanting surgeon. However, there is a high rate of agreement of 70%–86% between the procuring and transplanting surgeons, suggesting that forms filled out only by the procuring surgeon may be sufficient. An option could have been to only use organs with a Quality form filled out by both parties, but a part of the C2 organs are deemed not transplantable by the procuring surgeon. For these organs, no form is available of the transplanting surgeon. Excluding these cases, would therefore cause under reporting of the C2 organs. Second, the retrospective design of this study resulted in missing data regarding information that would have been interesting to investigate further. For example, no information regarding previous abdominal surgery was available, which could be valuable information to have prior to the procurement because of possible adhesions due to prior abdominal surgery. Also further classification of the type of injury was lacking for 60% of Quality Forms of kidneys and the necessity for repairment was limited available, since this information is not consistently captured. Also investigating whether *en-bloc* procurement of liver and pancreas leads to less injuries would be interesting, but since the number of grafts procured *en-bloc* number was relatively low, we did not include this is our analyses. One of the major strengths of this study is the mandatory nature of follow-up registries for kidney and liver transplantation in the Netherlands, ensuring nearly complete follow-up data. Although since the relatively small number of pancreases with procurement-related injuries transplanted, it is difficult to draw conclusions from this analysis.

This study demonstrated that procurement-related injury in transplanted organs does not affect long-term graft survival. It is important to emphasize that this comes with a certain bias; in these organs, the procurement-related injury could be repaired, and therefore, these organs could successfully be transplanted. On the other hand, procurement-related injury contributed to the discard of 4% (324/8,044) of procured organs: 135 kidneys, 79 livers, and 110 pancreases. Every organ lost for transplantation due to preventable reasons is one too many. Therefore, further research should focus on preventive measures against procurement-related injuries. We previously demonstrated that procedures during evening/night-time have a higher incidence of procurement-related injury than day-time procedures [22]. Centralizing the organization of organ procurement could also contribute to a decrease in procurement-related injuries. Although center volume was not specifically addressed in this study, de Boer et al. showed that centers performing more procurements had significantly fewer injuries (C1+ C2) for kidney and pancreatic procurement [3]. In 2023 Lam et al. suggested that cumulative sum (CUSUM) analysis plots with data from the Quality Forms could be of value to prospectively monitor procurement-related injury in a real-time manner, which could further lead to quality improvement and bring quality monitoring to a new level [23].

In conclusion, procurement-related injuries occur in 23% of abdominal organs procured in the Netherlands, resulting in 4% of the procured grafts not being suitable for transplantation. Despite this, the majority of kidney and liver grafts with procurement-related injury are still transplanted, showing no significant differences in 5-year graft survival compared with grafts with no procurement-related injury. This suggests effective decision making by transplant surgeons in determining the acceptability and reparability of procurement-related injuries. Auditing, national training of procurement surgeons, and certification contribute to this, and are important to even lower the incidence of procurement-related injuries in the future.

## Data Availability

The original contributions presented in the study are included in the article/Supplementary Material, further inquiries can be directed to the corresponding author.

## Abbreviations

BMI, Body mass index; CT, Computed Tomography; CI, Confidence interval; DGF, Delayed graft function; DBD, Donation after brain death; DCD, Donation after circulatory death; NTS, Dutch Transplantation Foundation (Nederlandse Transplantatie Stichting); eGFR, Estimated glomerular filtration rate; HR, Hazard ratio; KDRI, Kidney Donor Risk Index; MDRD, Modification of Diet in Renal Disease; NOTR, Netherlands Organ Transplant Registry; OR, Odds Ratio; PNF, Primary non function; SD, Standard deviation.
