# Supplementary material for: Ten Years of Quality Monitoring of Abdominal Organ Procurement in the Netherlands and Its Impact on Transplant Outcome
Source: Transpl Int. 2024 Jun 11;37:12989. doi: 10.3389/ti.2024.12989 (PMC11197516; doi:10.3389/ti.2024.12989)
Supplement: Supplementary file 1 [file DataSheet2.PDF]

Appendix 1: National protocol: Post mortem donor organ procurement[1]

4.6. Abdominal organ procurement procedures

Please be advised that organ dissection in the abdominal procedure is different for DCD and DBD donors. In DCD donors cold perfusion is started as soon as possible and any dissection is carried out after cold perfusion as described in chapter 4.3.2. In case of a DBD donor, dissection is preferably carried out as much as possible during the normal circulation of the donor, allowing for palpation of pulsations and to shorten cold ischemia time. The technique of mobilization is however comparable. The technique of standard DBD retrieval is described below:

4.6.1. Dissection of the avascular surfaces – organ mobilization

1. Pull the ascending and transverse colon to the left side and cut the posterior peritoneum starting from the distal part of the right external iliac artery up to the hepatoduodenal ligament (please be aware of the right ureter);
2. Perform extended Kocher maneuver by cutting the peritoneum at the right side of the duodenum. Also cut the avascular inferior border of the foramen omentale (Winslow). The pancreatic head from the right side has now been mobilized up to the right side of the abdominal aorta at the level of the celiac trunk and superior mesenteric artery;
3. Pull the small bowel to the left and upwards and cut the peritoneum between the right external iliac artery and ligament of Treitz;
4. Retract the mobilized ascending colon and the small bowel to the upper abdomen;
5. Free the anterior wall of the abdominal aorta and the inferior vena cava up to the upper border of the left renal vein before exploring the first 2-3 cm of the SMA for aberrant hepatic artery (consider the possibility of a right aberrant renal artery going to the right kidney on the anterior surface of IVC);
6. Mobilize the left renal vein. Free the posterior surface of the left renal vein from the abdominal aorta. It is not necessary to ligate and cut the left adrenal vein in every case;
7. If wanted (not necessary) cut and ligate the inferior mesenteric vein. In this way you can get good access to the celiac plexus, left renal and adrenal vein;
8. Ligation of the IVC is optional. Alternatively, if you plan to vent from the distal cava using active or passive suction, an extra ligature around the inferior cava can be placed. If necessary, ligate and cut the inferior mesenteric artery, it will give you more space and make it easier to cannulate the abdominal aorta;
9. Place colon and small bowel back into the abdominal cavity in the physiological position. Avoid colon and small bowel mesentery torsion – especially important during organ perfusion;
10. Mobilize the left liver lobe by incising the left triangular ligament;
11. Identify the left aberrant hepatic artery, if possible, prior to clamping of the supratruncal aorta. In case the aberrant left hepatic artery is absent, cut the lesser omentum (hepatogastric ligament) from the diaphragm down to the hepatoduodenal ligament. There is some fibrous tissue around the aorta and after cutting this tissue it is much easier to get around the aorta.

Be aware: sometimes dissection of the abdominal aorta may be impossible. For example in case of an obese patient with a left aberrant hepatic artery, which passes close to the diaphragm. In such

cases the aorta must be dissected and closed in the left thoracic cavity to optimize abdominal perfusion.

12. Perform examination of the hepatoduodenal ligament. Look for a right aberrant hepatic artery or right replaced hepatic artery, especially behind the common bile duct (CBD) and at the right side of the portal vein;
13. Identify and free 0.5-1 cm of the common hepatic artery (close to the gastroduodenal artery);
14. Identify and free 0.5-1 cm of the gastro-duodenal artery (close to the common hepatic artery and pancreas head);
15. Dissect and free the anterior side of the proper hepatic artery in the hepatoduodenal ligament;
16. Dissect and free 1.5-2 cm of the portal vein, starting 0.5 cm above the pancreas head and divide equally in case of both liver and pancreas transplantation;
17. Identify and dissect the common bile duct just above the pancreas head, free 0.5-1.5cm, ligate the distal part and cut the CBD;
18. Preferably: ligate the cystic duct. Alternatively, if you are not completely certain that you only have the cystic duct (and not CBD and/or right hepatic artery), the gallbladder should be opened, bile removed and the gallbladder cleaned with NaCl0.9% or Ringer's solution;
19. Flush the CBD and intrahepatic biliary tree using low pressure UW solution;
20. If arterial blood supply is very complicated and you are not able to recognize it retrieve the liver and the pancreas "en bloc". Please remember in rare cases, the pancreas may have to be sacrificed for the liver. In all cases, contact the transplant centers for consultation.

4.6.2. Abdominal perfusion in case of a DBD donor

Allow other teams to prepare the thoracic organs, if indicated.

1. Donor heparinization (heparin takes 3-5 minutes to start to work):
   - 300IU Heparin / kg donor body weight / i.v. (adults and children)
2. Fill the rapid perfusion system with cold preservation solution without air and close it with a clamp;
3. Prepare the inferior vena cava decompression system if desired by connecting a sterile thorax drain (22-24F) or fenestrated suction cannula with a long silicon tube to drain. Be aware of aberrant renal arteries and veins;
4. Decompression is obtained by cutting the vena cava above the diaphragm close to the right atrium, in consultation with thoracic surgeon in case of heart donation;
5. Reflect the colon and the small bowel to the upper part of the abdomen/thorax;
6. Ligate the aorta and the inferior vena cava close to their bifurcations;
7. First cannulate the abdominal aorta;
8. Second, the inferior vena cava;
9. Fix every cannula with thick ligature around vessels - avoid blood leakage;
10. Ligate with thick ligature or clamp off the abdominal aorta under the diaphragm;
11. Start cold perfusion of the abdominal organs;
12. Tie the proximal suture on the abdominal aorta to optimize abdominal perfusion and prevent abdominal preservation solution going into the thoracic organs. When using an aorta clamp for this, there is more risk for left liver lobe parenchyma injury;
13. Start continuous (external) topical cooling of the abdominal organs as soon as possible. Use for that cold, sterile Ringer's lactate or 0.9% NaCl solution and sterile ice or ice-slush. Replace the cold solutions regularly.

14. Check the quality of the abdominal organ perfusion, mesentery of the small bowel must be free of blood.
15. If necessary replace or reposition cannula of the aorta.

Be aware:

- In a DBD situation, the thoracic organs are usually procured first, because of shorter perfusion period and highest susceptibility of the heart to ischemic damage in the following order: heart and then lungs separately or heart and lungs together. Thoracic teams should however allow abdominal teams to extract the abdominal organs as soon as possible, at least within 60 minutes after perfusion.
- If no small bowel or pancreas is being retrieved, additional portal vein perfusion can be performed in situ.
- In 1-3% of the individuals, lower pole renal arteries arise from the CIA. In this case in particular, a common iliac artery should be cannulated below the lower pole artery. Additionally another tie must be put around the other CIA, for later closure, in order to avoid perfusion solution loss going into the left lower extremity. Ties should only be knotted and the CIA or aortic cannula inserted 35 minutes after administration of heparin (25,000 Units or 300 mg/kg BW).
- In case of severe arteriosclerosis of the abdominal aorta or CIA, the distal abdominal aorta may not be useable. In this case, the external iliac artery (EIA) should be used for perfusion access in order to avoid thrombo-embolisms or false cannulation. If the EIA is also not useable, the thoracic aorta could be used.
- Low pressure should be used in the portal vein in order to avoid sinusoidal damage.
- All retrieved organs are to be re-perfused on the back table.

4.6.3. Dissection during small bowel procurement (only in case of a DBD donor)

1. Perform resection of the ascending and transverse mesocolon to obtain free access to the superior mesenteric vessels (SMA, SMV).
2. Dissect and separate the SMA and SMV close to the uncinate process of the pancreas.
3. Transect the first jejunal loop at about 5-10 cm from Treitz ligament using the GIA stapler.

The entire intestine is kept in place in order to avoid traction on the superior mesenteric veins.

1. Transect the several small jejunal branches of the jejunal mesenteric artery close to the jejunal wall of the first loop.
2. Free the proximal parts of the mesenteric vessels for about 2 cm so the small pancreatic veins, joining the right part of the SMV, are ligated.
3. When the perfusion is ended, the liver-pancreas-intestine can be retrieved enbloc or seperately. In case of an isolated intestinal transplant, it may be necessary to prolong SMA and SMV using free venous and arterial grafts.
4.

Be aware:

- Antegrade decompression of the intestine as proposed by some centers is discouraged due to mechanical stress.
- Try to avoid tissue damage: pancreas (capsule, parenchyma) or mesenteric vessels
-

4.6.4. Simultaneous intestine and pancreas procurement (only in case of a DBD donor)

In the case of simultaneous pancreas procurement, inferior pancreato-duodenal vessels must be respected. Very early branching of the SMA may be a contra-indication for simultaneous procurement in rare cases and procurement of the intestine should be preferred. However, when

complex anatomical situations are present, en-bloc procurement with subsequent back table separation should be performed. The latter procedure shortens the donor procedure, but prolongs cold ischemia time.

4.6.5. Separate pancreas and liver procurement

1. Close and divide the duodenum from the jejunum with the Gastrointestinal Stapling Device (GIA);
2. Close and divide the duodenum at the level of the pylorus with GIA;
3. Divide hepatogastric and gastrocolic ligament close to the stomach wall starting from the pylorus up to the oesophagus ("shave the stomach"). Stay close to the stomach wall with your scissors. This is a safe way in case a left aberrant hepatic artery, arising from the left gastric artery, is present;
4. Reflect the stomach into the thorax;
5. Pull down the transverse colon and the greater omentum to achieve optimal visualization of the mesenteric vessels and the transverse mesocolon;
6. Cut the transverse mesocolon 3 - 4 cm beneath the lower part of the pancreas from the avascular transparent place, right from the middle colic artery, to the left side up to the spleen using a GIA. Mobilize the ascending colon;
7. Divide the root of the small bowel mesentery 3 - 5 cm below the uncinate process of the pancreas by using a gastrointestinal or vascular stapling device or ligation (try to do this in small steps);
8. Place the colon and small intestines on the legs of the donor;
9. Cut the gastroduodenal artery 0.3 - 0.5 cm from the common hepatic artery (mark pancreas side of the gastroduodenal artery with a Prolene 5/0 suture);
10. Cut the portal vein 2 cm above the pancreas head;
11. Free the common hepatic artery, the celiac trunk and first 5 mm of the splenic artery towards aorta;
12. Look for the dorsal pancreatic artery; in the absence of the dorsal pancreatic artery from the common hepatic artery or from the celiac trunk: Cut the splenic artery 0.3 - 0.5 cm from the celiac trunk, (mark the pancreas side of the splenic artery with a Prolene 5/0 suture);
13. Visualize the superior mesenteric artery and the celiac trunk from the right and the left side of the aorta;
14. Cut the patch from inferior vena cava with the left renal vein to fully expose the abdominal aorta;
15. Localize the ostium of the right renal vein(s) through the left renal vein opening (cutting) in the inferior vena cava wall;
16. Cut the VCI 1 - 1.5 cm above the ostium of the right renal vein;
17. Cut the ligatures placed on the aorta. Remove the cannula's and open the anterior wall of the aorta longitudinally. Stay in the middle;
18. Divide the aorta between the left renal artery and the right renal artery, leaving a patch for both arteries;
19. Cut the superior mesenteric artery with a patch from the aorta;
20. Cut the spleen ligaments and free the pancreas from retroperitoneal attachments up to the left side of the aorta. The pancreas should be handled using a no-touch technique: the pancreas should be held either by the duodenum or the spleen. Use the spleen as a "handle". Avoid touching and kinking of the pancreas.

Communication between the pancreas and liver teams is necessary in order to decide on the final use of pancreas for whole organ transplantation because of possible anatomical difficulties:

- In the presence of an **aberrant right hepatic** artery with a complete extrapancreatic course, one can decide to dissect this artery close to its origin with 1cm cuff or patch from the SMA (to be reconstructed to the donor GDA in the transplant center).
- In case of an **intra-pancreatic right hepatic artery**, its division should only be carried out after consultation between pancreas and liver teams. If this artery is transected proximal to the pancreatic head, the liver surgeon must have the possibility of implanting the right aberrant hepatic artery into the ostium of either gastroduodenal or splenic artery.

5. If the **dorsal pancreatic artery** is arising **from** the **common hepatic artery or from the celiac trunk** the procurement surgeon must communicate with the liver and the pancreas acceptor center(s). In these two cases of anatomical abnormality the common hepatic artery can be cut 3-5mm from the celiac trunk and the celiac trunkand the SMA can be given to the pancreas with the aorta patch, after consultation. In some cases (small children as donors and acceptors,difficult liver adult acceptor, no adequate "toolkit" for organ reconstruction) the pancreas procurement as a whole organ should be avoided.

21. Always procure the pancreas with the hilum of the spleen, for the typing laboratory cut only 3-4 cm of the external surface of the spleen;
22. Place the procured pancreas in ice cold UW until packaged;
23. Cut the aorta at the level of the proximal ligature;
24. Free the aorta with celiac axis completely from its retroperitoneal attachments;
25. Cut the diaphragm from the left side of the liver;
26. If the heart was not procured, cut the vena cava inferior 2 - 3 cm above the diaphragm and free it from pericardium. Gently lift the liver with a finger in the IVC during further dissection;
27. Cut the right anterior, lateral and posterior side of the diaphragm, going through the right adrenal gland. Place the liver in ice cold UW until packaged.

The detailed dissection of the liver hilus structures should be left to the transplant surgeon.

Be aware: Stay away from the liver ligaments to avoid liver capsule and parenchyma injury.

Remember: To always post-perfuse the liver through the portal vein, especially in case of:

- Suboptimal organ perfusion;
- Difficult aorta or iliac artery cannulation;
- Severe atherosclerosis;

The liver must be flushed on the back table with a minimum of 1000 ml cold preservation solution until a clear outflush is observed. The distance between the liver and the preservation solution container should not exceed more than 80 cm (normal pressure in the portal vein is about 6-12 mmHg).

4.6.6. Back table split of liver and pancreas after en-bloc removal

1. The separation of liver and pancreas starts with the division of the common hepatic artery and superior mesenteric artery.
2. Suture marking (towards the pancreas) of the gastroduodenal artery at the upper border of the head of the pancreas with consecutive transection of the GDA leaving a stump at the common hepatic artery.
3. The bloc is further divided by cutting off the splenic artery close to the celiac trunk. Following placement of an identification stitch (5/0 Prolene) at its distal part transection of the splenic artery in the middle of its origin and its first branch for the pancreas.
4. Transection of the portal vein well above the confluence of the splenic and superior

mesenteric vein will leave sufficient portal vein for the liver as well as for the pancreas (2cm above pancreas head).
5. An aberrant/accessory right hepatic artery might appear dorsally of the portal vein. (if present, transect the right hepatic artery at its origin preserving the SMA for the pancreas).
6. Ligation of the distal ductus choledochus and transection will end the division of liver and pancreas. At this time, the bile ducts should be rinsed without pressure and the cystic duct should be ligated (or, in case of uncertainty, the gallbladder should be opened and flushed).

4.6.7. Whole pancreas procurement for islet transplantation
The pancreas must be procured following the same rules together with duodenum (to avoid damage to the pancreatic ducts). Vessels don't need to be marked and can be cut shorter than in case of pancreas procurement for whole organ transplantation. Liver and pancreas should be removed separately or enbloc and be separated on the back-table.

4.6.8. Kidney – Separate kidney procurement
1. Cut the ligatures placed on the aorta and the VCI;
2. Remove the cannulas and open the anterior wall of the aorta longitudinally. Stay exactly in the middle;
3. Check the ostia of the renal arteries. Look for accessory renal arteries from aorta and from the iliac vessels;
4. Divide the posterior wall of the aorta exactly in the middle between the ostiae of the lumbar arteries;
5. Mobilize each kidney and the ureter with as much adjacent tissues as possible;
6. Cut the ureters close to the urinary bladder;
7. The right kidney has to be procured together with inferior vena cava up to the bifurcation (in case of multiple renal veins, a difficult recipient or a very short right renal vein, the inferior vena cava should be used for right renal vein elongation);
8. Each kidney should be placed in ice cold preservation solution until the organ is packaged using cold storage or connected to machine perfusion;
9. Reduce the adipose tissue around the kidney and the ureter and examine each kidney at the back table for: anatomy, state of perfusion, tumor, injury and infection.

4.6.9. Kidney – "en-bloc" kidney procurement on request
Remember: For donors < 5 years the kidneys should be removed "en-bloc", including complete abdominal aorta and IVC - from their bifurcations up to SMA and 1 cm above the renal veins (IVC). In the case of a pediatric donor always discuss your procurement technique with the kidney acceptor center(s).

1. After mobilization from retroperitoneum, the kidneys are removed starting at the lower end of the abdomen. Localize the ureters and cut the ureters close to urinary bladder. Mark the ureters with small mosquito clamps.
2. Pull up the ureters and the cannulas inserted in the IVC and abdominal aorta.
3. Cut the aorta and the IVC below the cannulas close to their bifurcations
4. Lift them gently up together with the ureters. Cut all tissues passing posterior to the aorta and IVC close to the ligaments and the muscles covering the vertebral bodies. This maneuver is continued upwards until the previous transections of aorta and IVC are reached.
5. Take out the kidneys and place the kidneys en-bloc in ice cold preservation solution until packaged.

Be aware: centralized preparation and preparation between the lower pole of the kidney and ureter is to be avoided (cave: blood supply of the ureter).
Finally, additional back table perfusion should be carried out to assess the perfusion quality and the vascular status of each renal artery. The venous outflow is to be assessed individually.
If necessary separate the kidney bloc on the back-table:

1. Remove the left renal vein from the IVC (with cava patch) to turn the specimen over.
2. Incise the posterior wall of the aorta in between the different lumbar arteries. This allows very easy definition of the orifices of (possibly aberrant) renal arteries.

The detailed dissection of the kidney structures should be left to the transplant surgeon.

Source:

1. Landelijk Overleg Uitname Teams (LORUT). National Protocol Post mortem donor organ procurement. 2022.

This protocol is the property of the Landelijk Overleg Uitname Teams (LORUT), organ advisory committee of the Dutch. Transplantation Society (NTV) and the Dutch Transplant Foundation (NTS).
For any questions or remarks concerning this National Protocol Post mortem donor organ procurement, contact the secretary of the Landelijk Overleg Uitname teams (LORUT):

Nederlandse Transplantatie Stichting
Haagse Schouwweg 6 | 2332 KG Leiden Postbus 2304 |
2301 CH Leiden transplantatiestichting.nl
