# Supplementary material for: Ten Years of Quality Monitoring of Abdominal Organ Procurement in the Netherlands and Its Impact on Transplant Outcome
Source: Transpl Int. 2024 Jun 11;37:12989. doi: 10.3389/ti.2024.12989 (PMC11197516; doi:10.3389/ti.2024.12989)

# Supplementary tables

**Supplementary Table 1 :** Kidney donor, recipient and procedural characteristics

|  | % Missing data | No procurement-related injury (C0) | Procurement-related injury (C1) |  |
| --- | --- | --- | --- | --- |
| Donor characteristics |  |  |  |  |
| Overall donor population |  | 1677 (71%) | 693 (29%) |  |
| Donor type   - DBD - DCD | 0% | 40%  60% | 39%  61% | p=0.64 |
| Age (in years) | 0% | 53 ± 16 | 54 ± 14 | p=0.18 |
| BMI (kg/m^2^) | 0% | 25 ± 5 | 26 ± 5 | **p=<0.01** |
| eGFR (mL/min/1.73m^2^) | 0.6% | 105 ± 45 | 103 ± 42 | p=0.42 |
| Gender, male | 0% | 53% | 58% | **p=0.03** |
| History of hypertension | 3% | 25% | 25% | P=0.94 |
| History of diabetes | 0.7% | 6% | 7% | p=0.24 |
| History of smoking | 0.3% | 57% | 55% | P=0.42 |
| Cause of death   - CVA * - Cardiac death - Trauma - Other** | 0% | 48%  16%  21%  15% | 49%  19%  20%  13% | p=0.28 |
| KDRI | 5% | 1.29 ± 0.34 | 1.29 ± 0.33 | p=0.98 |
| Recipient characteristics |  |  |  |  |
| Overall |  | 3025 (77%) | 909 (23%) |  |
| Age (in years) | 0% | 58 ± 14 | 58 ± 14 | p=0.81 |
| BMI (kg/m^2^) | 2% | 27 ± 11 | 27 ± 9 | p=0.90 |
| Gender, male | 0% | 63% | 61% | p=0.29 |
| History of diabetes | 8% | 26% | 25% | p=0.61 |
| History of cardiac disease | 2% | 15% | 15% | p=0.61 |
| Primary kidney disease   - Diabetes mellitus - Glomerulonephropathy - Renal vascular disease - Polycystic disease - Other | 0% | 13%  13%  10%  7%  57% | 11%  14%  12%  9%  54% | p=0.11 |
| Number of HLA mismatches   - 0 - 1-2 - 3-4 - 5-6 | 49% | 3%  27%  52%  19% | 2%  28%  53%  17% | p=0.58 |
| Procedural characteristics |  |  |  |  |
| Left kidney | 0% | 46% | 65% | **p=<0.01** |
| Number of arteries (% with >1 arteries)   - 1 artery - 2 arteries - 3 arteries - 4 arteries | 1% | 23%  77%  20%  3%  0,2% | 33%  67%  26%  4%  0,8% | **p=<0.01** |
| Number of veins (% with >1 veins)   - 1 vein - 2 veins - 3 veins | 0% | 92%  7%  1% | 93%  6%  1% | p=0.43 |
| Preservation method   - Cold storage - Machine perfusion | 0% | 39%  61% | 40%  60% | p=0.50 |
| WIT (in minutes), first period (only in case of DCD donation) |  | 16 ± 6 | 16 ± 6 | p=0.80 |
| WIT (in minutes), second period | 12% | 34 ± 15 | 34 ± 17 | p=0.46 |
| CIT (in hours) | 14% | 13 ± 6 | 13 ± 6 | p=0.96 |

*^Values are presented as mean (± SD) or percentage. KDRI = kidney donor risk index, donor-only^*

*^*Cerebral ischemia, intracerebral bleeding, subarachnoidal bleeding, subdural hematoma^*

*^** Suicide, Brain tumor, meningitis, status epilepticus, medical complication, epiglottitis/laryngitis, status asthmaticus, not specified^*

**Supplementary Table 2**: Multivariable logistic regression analysis and Cox regression analyses evaluating associations of donor, recipient, and procedural characteristics with the risk of delayed graft function and graft failure in the recipient. Results of model 3.

|  | DGF / OR | P-Value | Graft failure/ HR |  |
| --- | --- | --- | --- | --- |
| Surgical injury | 1.02 [0.81-1.31] | p=0.85 | 0.99 [0.75-1.33] | p=0.99 |
| Donor characteristics |  |  |  |  |
| Age | 1.01 [1.00-1.02] | **p=<0.01** | 1.02 [1.01-1.03] | **p=<0.01** |
| BMI | 1.02 [1.00-1.05] | **p=0.02** | - 1. [0.98-1.04] | p=0.75 |
| Gender (male as reference) | 0.72 [0.58-0.89] | **p=<0.01** | 0.84 [0.65-1.08] | p=0.17 |
| History of diabetes | 0.79 [0.51-1.23] | p=0.30 | 1.27 [0.81-1.98] | p=0.29 |
| History of hypertension | 1.58 [1.22-2.04] | **p=<0.01** | 1.08 [0.81-1.45] | p=0.59 |
| Cause of death |  | **p=<0.01** |  | p=0.36 |
| - CVA (reference) | 1 |  | 1 |  |
| - Cardiac | 1.11 [0.85-1.47] |  | 0.91[0.66-1.26] |  |
| - Trauma | 1.08 [0.81-1.43] |  | 0.76 [0.53-1.08] |  |
| - Other | 0.52 [0.39-0.71] |  | 1.04 [0.74-1.46] |  |
| DCD donor (DBD as reference) | 2.88 [1.23-6.71] | **p=0.02** | 0.83 [0.33-2.10] | p=0.69 |
|  |  |  |  |  |
| Recipient characteristics |  |  |  |  |
| Age | 0.99 [0.98-0.99] | **p=<0.01** | 0.99 [0.98-0.99] | **p=<0.01** |
| BMI (kg/m^2^) | 1.01 [0.99-1.01] | p=0.17 | 1.00 [0.99-1.01] | p=0.36 |
| Gender | 0.88 [0.72-1.01] | p=0.24 | 1.18 [0.92-1.50] | p=0.20 |
| History of diabetes | 1.50 [1.15-1.96] | **p=<0.01** | 1.25 [0.92-1.72] | p=0.25 |
| History of cardiac disease | 1.64 [1.23-2.18] | **p=<0.01** | 1.48 [1.08-2.02] | **p=0.02** |
| Primary disease |  | p=0.41 |  | p=0.07 |
| - Polycystic disease (reference) | 1 |  | 1 |  |
| - Diabetes Mellitus | 0.63 [0.37-1.09] |  | 1.12 [0.56-2.24] |  |
| - Glomerulonephritis | 0.84 [0.52-1.34] |  | 0.88 [0.47-1.67] |  |
| - Renal vascular disease | 0.76 [0.46-1.26] |  | 1.77 [0.97-3.25] |  |
| - Other | 0.71 [0.47-1.06] |  | 1.20 [0.70-2.04] |  |
|  |  |  |  |  |
| Procedural characteristics |  |  |  |  |
| Cold ischemic time | 1.04 [1.02-1.07] | **p=<0.01** | 1.04 [1.01 – 1.06] | **p=<0.01** |
| First warm ischemic time | 1.01 [0.99-1.03] | p=0.23 | 1.02 [0.99-1.04] | p=0.12 |
| Second warm Ischemic time | 1.00 [0.99-1.01] | p=0.98 | 1.00 [0.99-1.01] | p=0.73 |
| Multiple arteries | 1.12 [0.88-1.41] | p=0.33 | 1.10 [0.84 -1.43] | p=0.49 |
| Multiple veins | 1.38 [0.94-2.03] | p=0.11 | 1.21 [0.79-1.83] | P=0.40 |
| Left kidney (right as reference) | 0.93 [0.76-1.15] | p=0.52 | 1.03 [0.80-1.32] | p=0.81 |
| Preservation method (CS as reference) | 0.56 [0.45-0.71] | **p=<0.01** | 0.82 [0.63-1.06] | p=0.12 |

**Supplementary Table 3**: Liver donor, recipient, and procedural characteristics

|  | % Missing data | No procurement-related injury(C0) | Procurement related injury (C1) |  |
| --- | --- | --- | --- | --- |
| Donor characteristics |  |  |  |  |
| Overall |  | 77% (n=1011) | 23% (n=306) |  |
| Donor type (DBD) | 0% | 54% | 49% | p=0.08 |
| Donor age (in years) | 0% | 51 ± 16 | 51 ± 16 | p=0.73 |
| BMI (kg/m^2^) | 0% | 25 ± 5 | 26 ± 7 | **p=0.04** |
| Male | 0% | 51% | 57% | p=0.06 |
| History of hypertension | 3% | 23% | 25% | P=0.88 |
| History of diabetes | 0.7% | 5% | 3% | p=0.34 |
| History of smoking | 0.3% | 57% | 57% | P=0.95 |
| Cause of death   - CVA * - Cardiac death - Trauma - Other** | 0% | 54%  12%  19%  14% | 50%  13%  20%  16% | p=0.72 |
| Liver function   - ASAT - ALAT - Total bilirubin |  | 72 ± 97  59 ± 90  10 ± 7 | 75 ± 149  71 ± 220  10 ± 7 | p=0.66  p=0.16  p=0.26 |
| Recipient characteristics |  |  |  |  |
| Age (in years) | 0% | 52 ± 15 | 53 ± 13 | p=0.33 |
| BMI (kg/m^2^) | 0% | 25 ± 5 | 26 ± 7 | p=0.18 |
| Male | 0% | 65% | 66% | p=0.73 |
| Primary liver disease   - Acute hepatic failure - Benign liver tumours - Cancer (HCC, biliary tract) - Cholestatic/ biliary disease - Cirrhosis - Other | 0% | 3%  6%  29%  29%  23%  9% | 3%  4%  29%  29%  27%  9% | p=0.68 |
| Procedural characteristics |  |  |  |  |
| WIT (in minutes), first period (only in case of DCD donation) | 12% | 16 ± 5 | 17 ± 5 | p=0.71 |
| WIT (in minutes), second period | 4% | 34 ± 12 | 35 ± 13 | p=0.31 |
| CIT (in hours) | 0% | 7 ± 3 | 7 ± 3 | p=0.33 |
| Liver vascularisation   - Normal arterial anatomy - Aberrant vascular anatomy | 0% | 67%  43% | 62%  48% | p=0.07 |

*^Values are presented as mean (± SD) or percentage. KDRI = kidney donor risk index, donor-only^*

*^*Cerebral ischemia, intracerebral bleeding, subarachnoidal bleeding, subdural hematoma^*

*^** Suicide, Brain tumor, meningitis, status epilepticus, medical complication, epiglottitis/laryngitis, status asthmaticus, not specified^*

**Supplementary Table 4:** Multivariable Cox regression analyses evaluating associations of donor, recipient, and procedural characteristics with the risk of (death censored) graft failure in the liver recipient. Results of model 3.

|  | Graft failure/ HR |  |
| --- | --- | --- |
| Surgical injury | 0.92 [0.66-1.28] | p=0.61 |
| Donor characteristics |  |  |
| Age | 1.01 [1.00-1.02] | **p=0.04** |
| BMI | 1.02 [0.99-1.06] | p=0.23 |
| Gender (female as reference) | 1.29 [0.96-1.72] | p=0.09 |
| History of diabetes | 1.92 [1.16-3.18] | **p=0.04** |
| History of hypertension | 1.31 [0.17-10.42] | p=0.80 |
| Cause of death |  | p=0.10 |
| - CVA (reference) | 1 |  |
| - Cardiac | 0.68 [0.42-1.11] |  |
| - Trauma | 1.32 [0.90-1.94] |  |
| - Other | 0.99 [0.64-1.52] |  |
| DCD donor (DBD as reference) | 2.3 [1.19-4.64] | **p=0.01** |
|  |  |  |
| Recipient characteristics |  |  |
| Age | 0.98 [0.97-0.99] | **p=0.03** |
| BMI (kg/m^2^) | 1.01 [0.98-1.03] | p=0.39 |
| Gender (male as reference) | 0.72 [0.53 -0.98] | **p=0.04** |
| Primary disease |  |  |
| - Acute hepatic failure - Benign liver tumours - Cancer (HCC, biliary tract) - Cholestatic/ biliary disease - Cirrhosis - Other | 1  0.33 [0.13-0.88]  0.30 [0.15-0.58]  0.34 [0.18-0.65]  0.33 [0.17-0.64]  0.44 [0.22-0.89] | **p=0.02** |
| Procedural characteristics |  |  |
| - Cold ischemic time | 1.04 [0.99-1.09] | p=0.15 |
| - First warm ischemic time | 0.99 [0.95-1.03] | p=0.53 |
| - Second warm Ischemic time | 1.00 [0.99-1.01] | p=0.98 |
| - Aberrant arterial anatomy | 1.03 [0.76-1.39] | p= 0.85 |

|  | % Missing data | No procurement-related injury (C0) | Procurement-related injury (C1) |  |
| --- | --- | --- | --- | --- |
| Donor characteristics |  |  |  |  |
| Overall donor population |  | n=223 | n=10 |  |
| Donor type   - DBD - DCD |  | 68%  32% | 60%  40% | p=0.73 |
| Age (in years) |  | 32 ± 13 | 30 ± 10 | p=0.61 |
| BMI (kg/m^2^) |  | 23 ± 3 | 23 ± 3 | p=0.77 |
| eGFR (mL/min/1.73m^2^) |  | 123 ± 49 | 88 ±31 | p=1.34 |
| Gender, male |  | 46% | 40% | p=0.76 |
| History of hypertension |  | 8% | 0% | p=0.78 |
| History of smoking |  | 48% | 20% | p=0.33 |
| Cause of death   - CVA * - Cardiac death - Trauma - Other** |  | 45%  5%  31%  18% | 20%  20%  40%  20% | p=0.17 |
|  |  |  |  |  |
| Recipient characteristics |  |  |  |  |
| Overall |  | n=223 | n=10 |  |
| Age (in years) |  | 43 ± 8 | 43 ± 7 | p=0.92 |
| BMI (kg/m^2^) |  | 24 ± 14 | 22 ± 9 | p=0.53 |
| Gender, male |  | 53% | 60% | p=0.45 |
| History of cardiac disease |  | 9% | 10% | p=0.99 |
| Primary disease   - Diabetes mellitus type I - Diabetes mellitus type II - Type not specified |  | 91%  1%  8% | 100% | p=0.63 |
| SPK |  |  |  |  |
| WIT (in minutes), first period (only in case of DCD donation) |  | 5 ± 7 | 6 ± 7 | p=0.76 |
| WIT (in minutes), second period |  | 25 ± 8 | 25 ± 7 | p=0.94 |
| CIT (in minutes) |  | 506 ± 134 | 573 ± 42 | p=0.39 |

**Supplementary Table 5**: Pancreas donor, recipient, and procedural characteristics

Supplementary Figure 1: Death-censored graft survival until 5 years post pancreas transplantation, according to procurement related injury; any C1 injury (Log rank test p= 0.86)


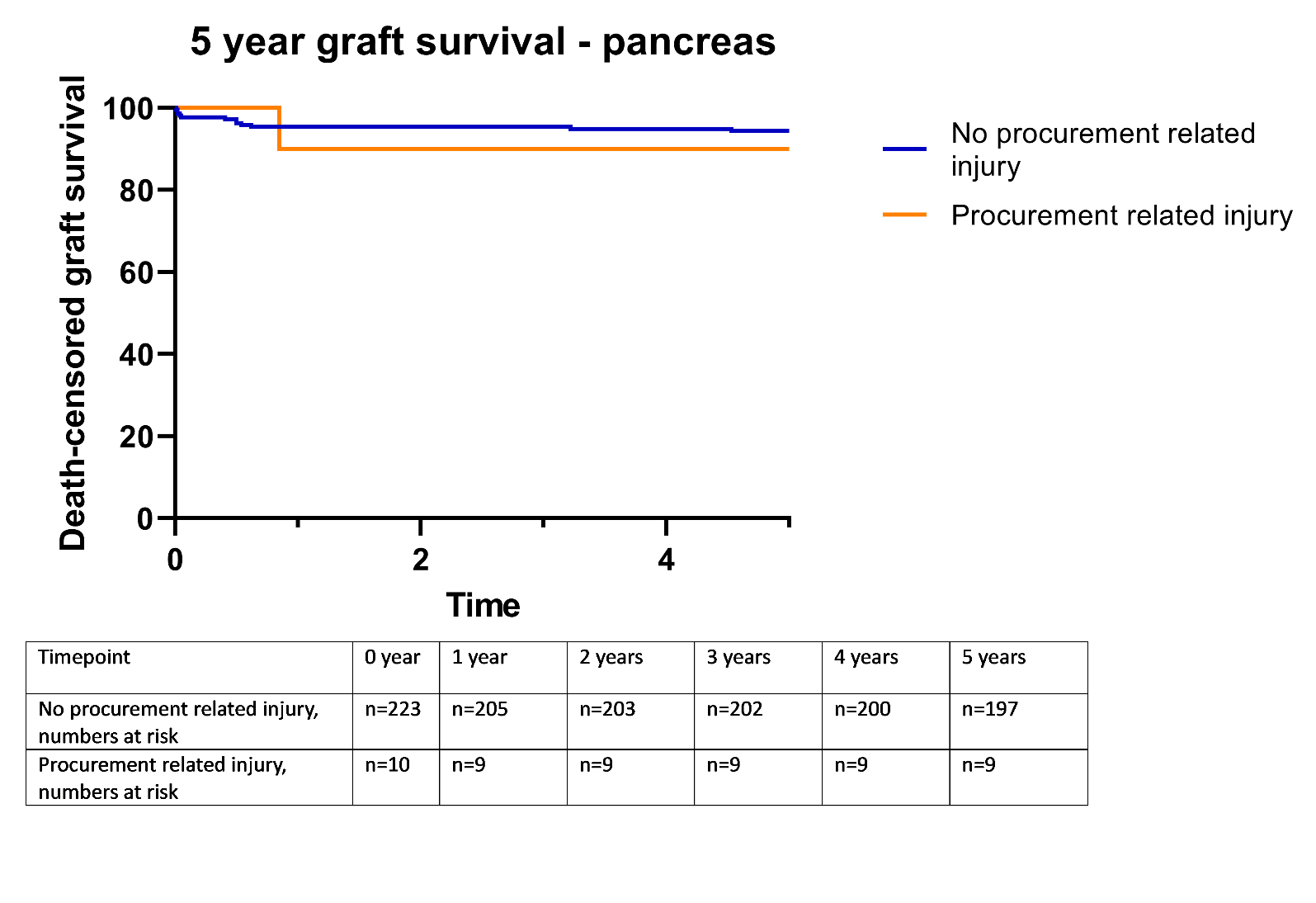

Supplement: Supplementary file 2 [file DataSheet1.docx]
